# Spotted Fever

**Published:** 1873-06-01

**Authors:** J. B. Weed


### SPOTTED FEVER.

BY J. B. WEED, M.D.

READ BEFORE THE CLARK COUNTY MEDICAL SOCIETY, APRIL 2D, 1873.

The so-called spotted fever, according to daily report, is still doing its fearful work in many locations, against all the skill of the profession. The prevalence of the disease as an epidemic indicates the presence of a special cause. What this special cause consists of, like the special cause of all of our epidemics, is not yet known; and we may not be able to determine, with our present means of investigation.

We may know some of the conditions on which the special cause depends for its operation. Any condition ascertained to be constantly *present*, preceding and accompanying the development of the disease, and, as constantly *absent* in persons not attacked, and in locations where the disease does not prevail, may be considered an essential condition, a link without chain of conditions, without which the chain would be broken ; the special cause, whatever this may be, become inoperative, and without which the disease could not become developed.

Such essential conditions are looked upon as the causes of disease, according to the extent of our knowledge and remedies directed to their removal in the cure and prevention of disease. It is especially in preventing disease that these remedies are efficacious. These essential conditions refer not only to the condition of the subjects of disease, which are more or less permanent or habitual, but also refer to the more transient physical and mental conditions which are dependent upon the immediate physical surroundings and moral influences.

An essential condition, consisting of a particular state more or less permanent or habitual, is spoken of by the profession as a predisposing cause; and the more transient conditions, dependent upon the immediate physical surroundings and moral influences, as the exciting causes of disease. It is thus we have two classes of causes — the first embracing the pre-existing conditions present, the second embracing the surrounding influences immediately attendant upon the development of the disease, each class being susceptible of subdivision, as our knowledge may extend.

Looking for the predisposing cause of this fatal malady in some condition of the system peculiar to persons in the localities in which the disease prevails, we find one constant peculiarity present in all the cases preceding and accompanying the development of this disease, and also as constantly absent in the locations where the disease does not prevail. The locations where the disease prevails are known as malarial districts, where malarial fevers, chills and fever, have always prevailed, or prevailed the season previous.

In these districts, not only those persons who suffer with chills and fever, but those who do not thus suffer, are affected with a chronic poisoning—a peculiar condition of the system known as malarial cachexia. This cachexia, when not the sequelæ of chills and fever, is a condition which is developed so gradually, the accumulation of the poison so insidious, that the system does not react against it in its effort, with chill and fever, to throw off the poison, but may do so on taking some tonic treatment to strengthen the nervous system, or on removal to some more healthful location.

This cachexia is manifest by the color of the skin, enlargement of the spleen and liver, neuralgia, etc. This condition has been found to consist not only of these derangements, but also of a peculiar condition of the blood—an absence of the normal amount of red corpuscles and the constant presence of a black pigment. This pigment, no doubt, is the debris resulting from the decay of that microscopic plant which has its growth in the system, and is now known as the *ague plant.*

This pigment in the blood accounts for the peculiar color of the skin, the neuralgia, and other nervous phenomena, and also gives a rational explanation of the well-known effects of remedial agents used in their removal.

This malarial condition is the only abnormal condition yet ascertained to be present in all these persons attacked with spotted fever. This pigmentary condition of the blood probably constitutes the co-operating or predisposing cause of this fearful disease.

The exciting causes appear to be any influence capable of disturbing, depressing, or transiently exhausting some portion of the nervous system, such as fear, anxiety, loss of sleep, fatigue, want of food, exposure to cold, etc. Exposure to extreme cold appears to have been the exciting cause in many cases. Such exposure would have a tendency to exhaust the very centers of the nervous system on which the disease locally manifests itself, the centers of sensation in the spine and base of the brain.

The intensity of the predisposing and the exciting causes, as factors in producing the disease, often bear an inverse relation to each other — a feeble cause exciting the disease in one strongly predisposed, while the gravity of the disease holds a direct relation to both, the special cause being always the same. The phenomena presented in each case has a definite relation to each of the causes producing the disease. The local tendency of the disease is impressed upon it by the special cause, which is the inflammation and exudation in the spine and brain.

In cases in which the predisposing cause is intense (a large amount of malarial poison in the blood), we have manifest malignancy, with the local manifestations absent, or but feebly marked, such cases terminating fatally before the local affection becomes developed. In cases in which the predisposing cause is feeble (small amount of malarial poison in the blood), the reverse takes place; there is an absence of malignancy, and prominence of local and inflammatory symptoms. In these inflammatory cases, the local tendency of the disease kills by destroying the function of the base of the brain which presides over the circulation and respiration.

In cases where both the predisposing and the exciting causes are in high degree of intensity, we have an explosive development of the disease ; a dying condition is developed instead of disease. In such cases there is so much poison so suddenly developed in the blood that this vital fluid is so far disorganized as to be unequal to the support of life; or the nervous system receives such a shock that the person is dying from the first.

The locations in which the disease prevails, the subjects it selects for its victims, the diversity of the phenomena presented by the disease, the absence of its prevalence in the centers of large cities, where ague does not prevail, except in imported cases, all point to this malarial condition of the system as the co-operating or predisposing cause of this terrible disease, spotted fever.

Concerning the physical character of the *special* cause we know nothing for a certainty, and, perhaps, had better not indulge in speculation. It no doubt pervades the whole country in its pestilential march westward, but can make its withering influence manifest upon such only as are brought into its deadly grasp by some predisposing condition of their systems; and these it still cannot reach without some accessory influence exciting the causes into action.

We see this exemption illustrated by those who are in a like condition in the afflicted locations, but who, by the systematic regulation of their lives and their sanitary surroundings, avoid the exciting causes.

The same immunity has been observed during the prevalence of the most pestilential visitations of disease in the priests who ministered to the spiritual and temporal wants of the sufferers, and walked amid the pestilence unharmed. These persons had cultivated, systematic habits, disciplined minds — were disconnected from the rest of mankind— were not depressed or exhausted by toil, care, anxiety or grief, and therefore avoided the exciting causes, enjoyed the immunity, and thereby the veneration, of those around them.

The special cause may consist of microscopic vegetable or animal forms, the spores of which float unseen in the atmosphere, from which they are taken into the system, and there finding the essential conditions of their short lives, soil, food, organic matter—not under the control of the vital forces—become developed in quantities sufficient to produce the disease; this pigment in the blood acting as the soil.

Or, it may consist of some imponderable agency — some of the yet undiscovered forms of electricity which obtains from planetary coincidence—and this, acting on the obsolete organic matter, determines chemical changes, thus disturbing the vital changes, and poisoning the fluids of the system.

The theory of vegetable forms is the more probable, as we know that each kind of vegetation has its definite relation to the different parts of the human organism, by which, when taken into the system, it disturbs the nutrition of the parts for which it has special affinity.

However this may be, what concerns most is its blighting effects. The cure and the prevention of the disease demands our attention. The treatment so far has not been flattering to the profession — unless we take into the account the cases which were imaginary—a large percentage being fatal, and the smallest number in which the treatment can be said to have turned the scale in favor of recovery.

In relation to a proper treatment, a few words will be sufficient. The appropriate remedies should be directed to obviate the danger. In inflammatory cases, in which the local tendency of the disease endangers the extinction of life, a certain amount of depletion will be found beneficial, and this should be adjusted to the degree of inflammatory action, and the danger from the progress of the local affection. Revulsive remedies should also be used locally, such as would act promptly, and with sufficient force to prevent, if possible, the exudation taking place to such an extent as to destroy the function of the parts involved, care being taken not to induce a fatal amount of prostration.

In the malignant cases, in which the danger is from prostration by want of nerve-power from blood-poison, the nervous system should be sustained by tonics and stimulants. The poison should be eliminated and neutralized by depurant and antiseptic medicines. The choice and efficient application of these remedies will depend upon the judgment of the physician, which has been matured by experience in the use of these remedies, while in possession of all the facts of disease.

With the best skill there must be great fatality, the local tendency being to parts the function of which is so absolutely essential to life, and because of the malignancy in the other class of cases.

In view of the fatality, the suffering which medicine will not even palliate, the small number of recoveries, and the infirmities of most of those who do recover, ought we not turn our attention to the prevention of the disease?

If it was the approach of cholera, the supervisors of the public health would be active; vigilant committees be appointed to daily visit private residences, to enforce all the hygienic and dietetic rules known to science; drainage would be perfected; all the disinfectants profusely employed; and all the sanitary regulations, known or imagined, rigidly enforced. This plague, worse than cholera, does not appear to move either the public or professional mind; and why not? Is it because it mostly attacks the humble in the rural districts? Every physician worthy of the calling will use his best efforts for all those for whom he is responsible. The disease *can be prevented by removing the predisposing cause* — by curing this malarial condition. The appropriate remedies will, in a few days, remove this condition, and prevent the possibility of an attack of spotted fever.

